# Twenty-seventh Annual Meeting of the United States Veterinary Medical Association

**Published:** 1890-11

**Authors:** 


### SOCIETY PROCEEDINGS.

*Twenty-seventh Annual Meeting of the United States Veterinary Medical Association* was held at the Auditorium, Recital Hall, Chicago, September 16th and 17th, 1890.

The Comitia Minora spent the morning session of September 16th in an executive meeting. The second session was called to order at 3 o'clock P.M. by President Michener. The following named members responded to the roll call by the secretary:

Drs. Adair, Atkinson, Barrow, T. S. Butler, Clement, Crowley, Crego, Wm. Dougherty, Eves, Faust, Faville, Hoskins, Howe, Huidekoper, Lemay, Liautard, Lyford, R. A. McLean, Marshall, J. C. Meyer, Sr., J. C. Meyer, Jr., Michener, Paquin, James B. Rayner, Thos. B. Rayner, James L. Robertson, Salmon, Trumbower, Turner, Weber, Winchester and Zuill.

There were also present a number of veterinarians, not members, from Pennsylvania, Ohio, Kentucky, Indiana, Michigan, Illinois, Iowa, Wisconsin, Missouri, Minnesota, Kansas, Indian Territory and South Dakota.

The President introduced Dr. W. L. Williams, of Bloomington, Ill., who delivered the following address of welcome:

### ADDRESS OF WELCOME.

MR. PRESIDENT AND GENTLEMEN: When we invited and urged you to convene this meeting here, our message bore an inferential promise of a hearty welcome, which it now becomes our pleasant duty to endeavor to fulfill. In a formal invitation it is scarcely possible to convey to a desired guest the full measure of the welcome which awaits him, nor to advise him wholly of the many reasons why a visit would be gratefully enjoyed, and so social custom has long decreed that an invitation be brief, and that many of the essential reasons for bidding the guest come, and for according a hearty greeting, should remain unexpressed until he has arrived at the house of the host, who, surrounded by the inspiring influences of home, may all the more fitly give voice to his thoughts. So, ere you enter upon the duties for which you have come in our midst, we would invite you to pause and briefly consider a part of the reasons why we extend to you a most hearty welcome, with the hope that they may gladden and cheer your labors. Were it my duty to welcome you specially to Illinois, the State of my birth, the scene of my efforts as a student and veterinarian, there would certainly be no lack of suitable reasons, with our vast live stock interests, the first in value of any State in the Union, the long list of serious contagious diseases which exist, or have existed, in our midst, their economic and sanitary importance, and the resulting large body of young veterinarians, greater in numbers than in any other Western State, who have every reason for heartily greeting you here for the good which may come to ourselves by commingling with you. It is not, however, to Illinois, but to the West and Northwest, that we bid you welcome; to the live-stock producing area of the nation, and one of the most important live stock regions of the world. You now visit for the first time the source of live stock supplies not alone to the nation, but to no small extent to several foreign countries.

Our meat-producing animals are rarely equalled, never surpassed, in any land for quality and richness, and our quantity is sufficiently great that after supplying the wants of the nation we still have an enormous supply of meat and dairy products for exportation. We breed and supply to home and foreign markets a vast number of horses, among which are the speediest pacers and trotters and as fleet thoroughbreds and as powerful draft horses as are to be found in the world. In building this comparatively recent live stock interest, every valued breed of every land has been drawn upon for its choicest individuals, and yet we have numbers and, value considered, the healthiest live stock population in the world. This being the fountain-head from which the nation's live stock supplies are to be drawn, the freedom of this area from serious diseases has an inestimable value from a monetary and sanitary standpoint, and directly influences the health and wealth of the nation. To no one can these questions prove of more direct interest than to our profession, and especially to your society, which, as now constituted, is largely made up of veterinarians representing that part of the country which consumes a large part of our live stock products.

There must be a strong reciprocal interest between the older and better organized profession of the East and the younger and now numerically equal body of Western veterinarians. Although the growth of our profession throughout the country has been rapid almost beyond belief, you are now, for the first time, in the midst of the most phenomenal part of the entire body, which practically the creation of the past decade has, within ten years, increased by nearly or quite 1500 per cent., a growth heretofore unequalled in any profession in any land at any age. This young, rapidly grown and, to some extent, poorly equipped veterinary profession must in the future be, to a great extent, the guardian of the health and worth of this vast animal population, among which widespread and fatal disease would mean financial ruin to those engaged in breeding and rearing live stock, would cripple one of our important sources of national wealth, and through those diseases communicable from animal to man, or those which render the flesh of affected animals unfit for human food, would jeopardize the health of the whole people.

Recently, we were astonished and alarmed to find that a serious outbreak of pleuro-pneumonia existed in this city, from which great numbers of live cattle are shipped not only to all parts of the nation, but to many foreign countries. As this insidious and destructive disease could not be tolerated here, the State at once took measures to suppress and eradicate it, and the nation, in due regard to its own interests, relaxed for a time its work with other infected areas of importance, and heartily joined the State in the prompt and now apparently effectual eradication of the disease at this peculiarly vulnerable point. So, when in 1887, equine syphilis was reported as existing to a serious extent for the first time in any English-speaking country in one of our principal horse-breeding centres, from which we annually export large numbers of valuable breeding animals to every part of the nation, there was a demand and necessity that it be controlled and extirpated, and, as a result, it has not been seen nor heard from since in that section.

In this region, during the past few years, the cattle have suffered more extensively from actinomykosis than perhaps in any other country, and the State Board of Live Stock Commissioners of Illinois, having recently taken the stand that the flesh of animals so affected is unfit for and dangerous as human food, we will soon witness in our courts one of the hardest fought legal battles in the history of veterinary sanitary science, one which will probably be quoted in the future as a precedent in dealing with this disease.

We young veterinarians, with these vast responsibilities awaiting us, are afforded the first opportunity of socially intermingling with many of the oldest and most favorably known veterinarians in America which, with the pleasure of meeting again many of those with whom we parted when leaving college, will amply repay us for laying aside for a few days our routine of every-day life.

We are offered, for the first time, an opportunity to listen to and profit by a series of papers representing the best thought of the oldest and best known section of our profession.

You are more cordially welcome, however, because you now offer us fellowship in your society in a practicable, available, desirable manner, in a way by which we hope to be able in the near future to repay you by adding numbers and interest to this society.

College selfishness, foibles, likes and dislikes have been partly blunted and overcome by our own State societies, but their influence being limited by the boundaries of States, we have now come to desire and fully realize the need of a central organization about which to rally a body which we may look upon as representing our highest ideals of thought and professional conduct.

We are grateful to be now offered an opportunity to sit with you in council; to become a part of your organization.

Comprising the numerical half of our profession in the United States, it will be conceded that we should constitute a part of the national organization, and we are glad that there is every promise that we will to-day, in proportion to our age and ability, assume a due proportion of the labor and responsibilities of the United States Veterinary Medical Association, and contribute what we can to round out its national character, that it may henceforth be viewed, not as a society of the Atlantic States, but as representing the best elements and thought of the veterinary profession of the nation.

This meeting seems to bear with it an inexpressed covenant that your conventions in the future are not to be kept within the former narrow limits, but are to be confined only by the national boundary lines. What a change from the past, when we may journey happily together across the Mississippi, over the Missouri, and beyond the Rocky Mountains, and convene a much larger assembly than this amid the orange groves on the shores of the Pacific. Such a consummation can scarcely be called visionary when we remember the rapid development and growth our profession is undergoing, and until this or other association shall cover this entire territory and include as members a fair proportion of the veterinarians of every section, we can not be said to possess a truly national veterinary society.

We look upon this meeting as making a beginning of a revolution in your organization, a revolution which, it is hoped, will bear you, and with you our whole profession a long step forward.

To-day you have convened this meeting doubly as far from the Atlantic coast as in any previous case; you will probably vote upon the largest list of applicants for membership ever presented at one time, look upon more new faces than ever before, and propose to make an important and sweeping change in your manner of admitting members.

You open your doors rather abruptly to a large company of strangers, mostly young veterinarians with limited experience in veterinary associations, thus offering to exchange important responsibilities with western veterinarians.

Heretofore the few members which you held in the West have apparently laid you under no responsibilities to this vast section, and in counting your effective forces or your liabilities or duties we have never figured as an essential factor, nor have we, as western veterinarians, ever felt that the character of your society, its objects or aims, its virtues or shortcomings, its joys or trials, were of any concern to us.

Henceforth we are to mutually share its duties and benefits, its trials and triumphs, its responsibilities and pleasures.

Western veterinarians are ready and anxious to take their proper place in your society as a part of a national organization, and meet you in fair number to-day to consummate this union. You perhaps wished to see more present, and certainly it would have pleased us, but after all, like new recruits to an army, before such accessions can prove a help and strength to this society they must learn what and how to do; they must be organized and trained; their forces concentrated, and all thoroughly amalgamated, so perhaps the not very heavy attendance may bode no ill to you. We hope, however, that those offered may rapidly train into able and willing workers, ever ready to advance the association towards its highest ideal, and through it to be of value to our whole profession.

From these new members we trust you will succeed in selecting able and willing representatives in every Western State, who will in the future see that the interests of the society shall no longer suffer from neglect, and that we shall hereafter furnish our due proportion of members, labor and thought.

When, however, you have secured ample membership in the West, have become intimately acquainted with a large part of our western veterinarians, have procured among us sufficient competent representatives to look closely after the general work of the society, have all your members under thorough discipline, and have held large and well attended meetings in every section of the whole country, you may yet fall short of the highest state of honor and usefulness of such an association.

There has been a phenomenal development of the veterinary profession in the West within the past ten years, from a few scattered representatives, mostly foreign born, to more than 1000 regularly qualified veterinarians.

Above and beyond all other reasons we welcome you here in the hope that your presence among us and our amalgamation with you will inspire the mass of this young and rapidly-growing part of our profession to higher thought, to deeper study, to rapid, firm, enduring progress.

The outlook here for earnest, competent veterinarians grows brighter every day. The general public is rapidly realizing his worth from an economic and humanitarian standpoint in the management of ordinary and everyday accidents and ailments, and the State and nation are rapidly discovering the value and need of our profession from a national economic and sanitary view in controlling and eradicating those contagious diseases of animals which so often ruin the owner and cripple the finances of the community; or through other diseases which in addition render the flesh of affected animals unfit for or dangerous as human food, or in that long list of diseases which are transmissable through the flesh or through contact from animal to man—usually of a very serious and deadly character.

The social standing, the emoluments, the honors to the competent veterinarian, must advance higher and higher at a rapid pace, while the incompetent, listless practitioner must find his room becoming more and more pinched and unsatisfactory.

We have at present in America but few prominent veterinarians who are really accomplishing something to elevate our profession; so few that they can be counted almost in a moment. Let them work as hard as they may they can accomplish little towards placing our profession on a level with other learned professions in this country, or with the veterinarian profession in many other lands.

We hopefully look to your society as the vital force, and to this day as the birthday which shall place a whole army of earnest veterinarians in this wide field for observation, research and thought, who, pressing forward harmoniously as one man, may yet, during the lives of most of us, place our profession on an equality with the veterinary or other scientific professions in any land.

More strongly than in any words we can command, we hope you will find in the cordial greetings of my Western colleagues, in their earnest, respectful attention to your deliberations, in their willingness and anxiety to make your stay among us as pleasant as is possible with our imperfect, hurried preparations; so cordial and hearty a welcome that you will remember this meeting, the West and Western veterinarians only with pleasure, and will find therein an irresistible invitation to come among us again at an early day.

President Michener responded to the address of welcome on behalf of the association as follows:

*Gentlemen of the Association, and particularly of the West:* There is a great difference between responding to an ordinary speech of welcome and to such a one as has been tendered us by Dr. Williams, and I fear that I cannot fully voice the appreciation of my associates from the East.

Dr. Williams has said so much, and has uttered it so sincerely, that we would be ungrateful indeed and unmindful of a pleasant duty if we failed to thank you, one and all, for our reception. We have some of us seen for the first time what is to us the great West, and I feel constrained to plagiarize a prominent man, and repeat that verily had Adam and Eve been placed in this section of our country, when expelled from the Garden of Eden, they would have raised their eyes reverently to heaven, and thanked God for the change.

Dr. Williams has given us a good idea of the extensive field for veterinarians in the West. If I can see aright, it is in the West that we are to look for our greatest progress. It is here that disease exists upon a great scale—diseases that we do not see at all, or but seldom, in the East, but which you in the West have successfully met. We may arrogantly assume that, as of old, the wise men are in the East, but I must insist that the veterinary profession is an exception, and that our brightest, most enterprising men are not content to remain within the narrow confines offered by our Eastern States. Greatness requires room, and it is here obtained. State universities of the West offer better inducements and more aid than do those of the East, as is evidenced by the veterinary departments of some Western States that compare most favorably with our exclusively veterinary colleges of the East. Let me cite an example of what the West does for its veterinarians by simply calling attention to our friend, Dr. Paquin. Who, East of the Alleghenies, has done so much? If, indeed, we except Dr. Salmon, who of us has accomplished any original work?

Perhaps I should have said that to-day we place the corner-stone; the foundation of our association has been building since June, 1863. That the association has not grown faster is due to the fact of the few artisans employed. With united, harmonious and well-directed work, our building should be completed, its happiest aims achieved and much and lasting good done the profession.

We will now receive the report of the Secretary, who will read the minutes of the last meeting.

Secretary Hoskins read the minutes of the twenty-sixth annual meeting of the association, as also the minutes of the meeting of the Comitia Minora held at that time, all of which were approved as read.

There being no unfinished business, the president called upon the Secretary to read the minutes of the meeting of the Comitia Minora held September 16th, 1890, and the same, as read, were approved.

Secretary Hoskins—The Comitia Minora recommend that the following names be dropped from the roll of membership for non-payment of dues.[1]

The report of the Comitia Minora was accepted, and the recommendation to drop the names of the delinquents from the roll was approved.

President Michener—There is a further recommendation of your Comitia Minora, gentlemen, and it is for you to say what action shall be taken in respect to the expulsion of Dr. Billings, which has been unanimously recommended. I will ask the Secretary to read the letter from Dr. Billings, so you may know the tenor of it, and if you feel, as your Comitia Minora did this morning, I do not think there is any doubt but what the name of Dr. Billings will be dropped from the list of members of this association.

[1] Published in the October number of the JOURNAL OF COMPARATIVE MEDICINE AND VETERINARY ARCHIVES.

Dr. McLean—I rise to a point of order. Our executive committee is established for the purpose of keeping out public clamor, such as this, from our general meetings. The letter referred to is utterly unworthy of any man, much less a veterinary surgeon. I move you that the reading of the letter be dispensed with. Seconded.

President Michener—I am willing to suppress the letter, if the associatjon wish to endorse the action of the Comitia Minora without hearing the letter read.

The motion to dispense with the reading of the letter referred to was unanimously carried. Om motion, the action of the Comitia Minora recommending the expulsion of Dr. Billings was approved, and Dr. Billings was declared expelled from membership in the association.

President Michener—The next recommendation of the Comitia Minora refers to the names recommended for admission to membership with us. The secretary will please read the names of those recommended by the Comitia Minora.

Secretary Hoskins read the names of the proposed members, and on motion of J. C. Myers, Sr., of Ohio, duly seconded, the names presented by the Comitia Minora were accepted collectively.

Mr. Faust—I move you, sir, that the secretary of the association be instructed to cast the ballot of the association for each of the members recommended for membership. Seconded. Carried.

Secretary Hoskins—In pursuance to the direction of the association, I have cast the ballot of the association for each of the candidates for admission to membership, and the following have been duly elected members of this association.[1]

President Michener—I think we older members would like to have the newly-elected members arise in their places, that we may know them.

In response to the suggestion of the chair, the newly-elected members arose in their places, and were complimented by President Michener as follows: I must say, gentlemen, that you represent the beauty of this national association. [Applause.]

Secretary Hoskins—The association has refused to recommend for reinstatement the name of Dr. W. T. McCoun, of Oyster Bay, Long Island.

President Michener—In that case there is no action for this body to take now, as the Comitia Minora have declined to recommend his reinstatement. Of course, any individual member has a right to find out from the Comitia Minora the reasons for their refusal, which, if deemed insufficient, the matter may be presented at a subsequent meeting of the association, and when the doctor can be present to defend himself. That is all the action we can take in the matter now. I will call for the report of the committee on intelligence and education, of which Dr. Austin Peters is chairman.

Dr. Peters submitted the report of the committee through Secretary Hoskins, as follows:

[1] Published in the October number of the JOURNAL OF COMPARATIVE MEDICINE AND VETERINARY ARCHIVES.

### REPORT OF THE COMMITTEE ON INTELLIGENCE AND EDUCATION.

MR. PRESIDENT AND GENTLEMEN: As chairman of your Committee on Intelligence and Education, I have the honor to submit the following report:

Last Autumn I placed myself in communication with the other members of this committee, and this Summer I sent the following circular to the assistant State secretaries:

23 COURT STREET, BOSTON, *July 10th, 1890.*

DEAR DOCTOR: Will you, as an assistant State secretary of the United States Veterinary Medical Association, kindly inform me as to the following points:

Have you any laws in your State protecting veterinary surgeons in the practice of their profession?

If there are any such laws, how do they work, and of what benefit are they?

Any information you may have to impart concerning the standing of the veterinary profession in your community; any recent advances it has made which will be of value in our report, or any hints or suggestions you may have to make concerning the work in hand, will be gladly received.

Yours truly,

AUSTIN PETERS, M.R.C.V.S.,

*Chairman of Committee on Intelligence and Education.*

From the various letters which I received in response, I have been able to compile the following rambling remarks. In sending the circulars to the assistant State secretaries, I took the precaution to enclose a stamped envelope with my address printed upon it, in order to be sure of a reply; yet out of a total of twenty-eight circulars I received but twenty answers. It seems to me that there is very little excuse for such lack of interest in matters pertaining to our profession, and it is my intention to furnish our president-elect with a list of the delinquents, in order that he may, if he sees fit, appoint men in their places.

From the view I take of the various matters considered in our report, the subjects of veterinary education, and what may be called intelligence, are so closely associated together that it is impossible to entirely separate them however, they will have to be considered to a certain extent as a whole.

I prefer, however, to begin with the subject of education, following with other topics which may be of interest to us. In 1863, when this Association was first organized, it consisted chiefly of men who were non-graduates; there were very few members of the Association who were graduates of veterinary schools, because there were no veterinary schools in the country from which they could graduate, and the few having diplomas obtained them by pursuing a course of study abroad. Yet these non-graduates were, as a whole, honorable, conscientious men; good practitioners of their profession; fairly well-read, and enjoyed the respect and confidence of the communities in which they lived. But a change has gradually taken place in the *personnel* of our Association; many of the old non-graduates have passed away and but a few remain; and the members who have joined us of late years have all been graduates of veterinary schools, and to-day it would be an impossibility for a non-graduate to become a member of our body. This change, however, was brought about in a great measure by these non-graduates themselves ; they said that now the country has a number of veterinary schools, there is no excuse for a young man about to enter the veterinary profession not acquiring an education to fit him for the work which he is to pursue in the future. Because they had to acquire their knowledge from the teachings of their fathers, from their readings and personal observations and experiences, was no reason why another generation should do so when it could have the advantage of schools, with instructors trained to work, dissecting rooms, laboratories and modern text books at their disposal, together with the advantages of hospital practice and clinics. These men asked for something better and they got it. The question now propounds itself to us, shall we be satisfied with our veterinary schools as they are ? or, shall we not in this great age of progress ask for something better ; a longer, more thorough course of study, with a higher educational qualification for matriculation, and a higher standard of graduation than at present prevails ? The old-fashioned non-graduates considered it no reflection upon themselves when they insisted that the present generation should make the most of advantages which they did not have, neither need we feel that we offer any affront to ourselves when we ask for something better.

I will not speak of individual schools, as it might lead to personalities and comparisons which are, to say the least, disagreeable ; but we do insist upon a higner education, a proper matriculation examination, a longer course of study in most of the schools—three sessions of nine months each, at least ; four sessions equally long would be better. The faculty should contain a large enough number of veterinarians to prevent the school graduating students with but one man's ideas. Where a number of veterinarians are interested in the school the students also receive greater clinical advantages, as in such instances practitioners will take pains to send their interesting or unusual cases to the school for the benefit of the students.

The matriculation examination at most of the veterinary schools on the Continent, where such an examination is required, is a farce, and men enter and graduate who can barely write their own names, much less read or write intelligently. This state of affairs should be remedied at once. and no student allowed to enter who did not, at least present evidences of a good English education, and the higher above this the better. A collegiate or academic course of some kind prior to the student's entering upon his veterinary studies should be encouraged, as it is to such an extent in the medical profession ; and to my mind the best preparatory course for the future veterinary surgeon is to attend one of our agricultural colleges ; here the study of botany, chemistry, agriculture, the characteristics of the various breeds of animals and general principles of breeding, together with sufficient knowledge of French and German to be able to read foreign veterinary and medical works, is one which will fit him for his future work.

Dr. M. R. Trumbower, of this committee, writes as follows : "It becomes daily more and more evident that we need a better and higher educational standard, and increased facilities in our veterinary schools, and

I think the inauguration of a longer and more scientific course of study is near at hand. I doubt, however, whether all the colleges can be induced to adopt a uniform standard for study and examination ; hence our support should be given to such institutions only which indicate a willingness to meet the advanced demands.

"It is absolutely necessary that the requirements for admission (educationally) be made sufficiently strict to exclude many such as were able to matriculate in the past. The professional standard can be raised by a representation of better men, and it is the only way to do it."

I heartily concur in the above, and agree with Dr. Trumbower that on this great continent there will always be veterinary schools and veterinary colleges, and that in the near future we, as an association, will have to countenance those only which evince a disposition to meet the requirements of the times.

Dr. Colsson, of this committee, refers to the fact that many veterinary graduates are unable to write a prescription properly, and even show a deplorable ignorance of the meaning of the signs and symbols used in prescription writing. He proposes to remedy this by adding an examination in pharmacy and prescription writing to the matriculation examination of the schools. I do not agree to this, as I think that the course of study in the veterinary schools themselves should deal with and remedy the defect.

In an article entitled "Veterinary Education in America," which appeared in the *American Veterinary Review* for January, 1890, Dr. Tait Butler, of this committee, has said much which I have left unsaid, and I can only add a fervent amen ! to what he writes.

In addition to educating the student, we must pay a little attention to the advancement of the practitioner. Because a young man has obtained his degree as a veterinarian, it is no reason for selling his library, and as a general rule he does not know any more the day after he graduates than he did the day before. A diploma is simply a certificate of having gone through a certain course of study, and successfully passed a required examination ; it merely marks a narrow landing on the long stairway of life ; and if a man's future is to be a success in the greatest measure, he must continue to be a diligent student and careful observer. Every veterinarian *must* keep abreast of the medical advances of the times ; he should take both of the American veterinary journals, one of the English veterinary magazines, and if he can read French or German, he should take a French or German veterinary magazine, or both, as the case may be. In addition to this, he ought to subscribe to a first-class medical periodical in order to know what is being done in the sister profession. He should not only subscribe to these journals, but he should read them carefully and intelligently after they come.

Because your teachers or professors told you a thing was so when you were a student, fifteen or twenty years ago, do not think it is so to-day, or that your instructor would say it was, if he has kept up with the times ; for in many ways medicine has made great advances during the past decade or two, and most writers on pathological subjects are ready to acknowledge that many things which they wrote a few years ago are wrong to-day.

The great discoveries of Pasteur, Koch and other investigators have revealed new truths as to the etiology and character of many diseases, which even if suspected by some, remained unproven until the modern methods of studying bacterial maladies were introduced. The progress made in the application of antiseptic and aseptic methods in surgery is also of quite recent origin, together with the knowledge of the parts played by different germs in the various suppurative and septic processes.

Our veterinary text-books are way behind the times on all these ancient discoveries, and will have to be re-written in the near future, or their places will have to be taken by more modern works.

The chairman of the committee last year in his annual report spoke of the tendency in medical schools to substitute recitations from standard text-books for the didactic lecture, the students studying up a chapter or two in various text-books daily, and then having recitations on what they had read. I am of the opinion, however, that the didactic lecture should remain a feature of the course in our veterinary schools, for the reason that our text-books are not up to the times, and that in most of our veterinary schools a cram course is given, and in no other way can so much be crammed into a student in the least possible space of time as by the didactic lecture.

As a student, I was very fond of listening to lectures, much preferring them to reading. A well-arranged course of lectures can be compared to a boy with a plum cake having his nurse pull out the plums, one by one, and feed them to him ; while a student reading can be compared to the same boy without a nurse, he having to overhaul a good deal of cake to get a few plums—the plums being the useful and interesting facts, the rest of the cake being the extraneous matter which it may be a waste of time for the student to read, and yet which he will read if his efforts are not wisely directed.

Before proceeding further, I wish to make another criticism on members of the profession. It is the readiness with which some men read articles written for the daily papers on scientific medical subjects, and accept them as valuable facts ; such as arguments against the germ theory of diseases, and the like. Remember, that the "doctors" who write for the newspapers are men who have no easy access to the columns of current medical literature, and that their opinions and arguments carry very little weight with members of their own profession ; it would be much better for the eager seeker after truth to confine his scientific reading to veterinary and medical magazines of acknowledged good character and leave newspaper science for the perusal of the laity—if members of that great body choose to waste time reading such articles.

Another factor for educating the veterinary practitioners is the veterinary association. Most States where there are any number of veterinarians now have their veterinary societies ; some States have more than one, and in some few instances certain State associations have combined with their colleagues in neighboring States to form interstate associations. These organizations are capable of doing much good to the profession in various ways. If members will take the pains to write good papers for the meetings, these essays and the discussions following them are of benefit to the practitioner from an educational standpoint. The careful reporting of interesting cases occurring in the practice of different members is also of value in this connection. In addition to the above, it should create a feeling of good fellowship and *esprit de corps* in the profession, and also form a valuable basis for co-operation when any work is to be undertaken for the advancement of veterinary science.

Every veterinarian who takes any interest in his work beyond the dolla's and cents he can make out of it, should belong to the local veterinary society where he resides, as well as to the great national organization meeting here to-day and to-morrow. In joining a veterinary association the practitioner should not join from a selfish motive for his own personal aggrandizement to advertise himself in any way, or to use it as a lever to gain a political position, but he should enter for the purpose of becoming better acquainted with his colleagues, adding to his education and doing all he can in a generous, unselfish way for the advancement of a noble and useful profession.

The next point of interest to be considered are the results of the circulars sent to assistant State secretaries asking them for information concerning legislation to protect the poor, oppressed, down-trodden veterinarians in different States, and the effect of such legislation upon his well-being, besides some of the interesting incidental matters which were brought out by the replies I received. As far as I have been able to ascertain, there are but four States in the Union where laws have been enacted regulating the practice of veterinary medicine, and in all of them the law is quite similar. These States are New York, New Jersey, Pennsylvania and Wisconsin. A description of the law in one State will answer for all, so I take the privilege of reading the letter on the Wisconsin law by Dr. V. T. Atkinson, of Milwaukee, Assistant State Secretary for Wisconsin, following with comments upon the letters of the Assistant State Secretaries in the other three States. The following is Dr. Atkinson's letter:

MILWAUKEE, August 25th, 1890.

AUSTIN PETERS, ESQ., M.R.C.V.S.,

DEAR DOCTOR: Yours of July 10th received in my absence, hence the delay in answering. In reply,

Chapter 347 of the Laws of Wisconsin for 1887 is, I am told, a copy of a law which was in force in New York at the time our law was enacted. It provides that no person shall be considered a veterinary surgeon nor allowed to give expert testimony as such or to collect fees for services by process of law except their credentials be recorded in the Veterinary Register, kept in the office of the Register of Deeds, of the county in which the practitioner resides. Such credentials may consist of, 1st, a diploma from a regular college; 2d, a certificate of membership from a duly organized veterinary medical association; 3d, an affidavit of five years' practice prior to the passage of the law.

In its workings this law is practically no good. Our State society has degenerated into a society of empirics. At the last meeting there were only three graduates present, of which I was one, and I remained only long enough to sever my connection with it.

You ask what the standing of our veterinarians are. It would be hard to strike an average, but I think the graduate, as a rule, commands the respect of the communities in which he resides, and that the profession is gradually assuming the important position to which it is entitled.

Concerning *protective* laws, my observation does not lead me to believe that they are likely to be of much service to any practitioner who is worth protecting. It has always seemed to me that the man who could not compete with a quack successfully would not be likely to reflect much credit on his associates in the profession even if the quack were removed from competition with him.

Hoping that I may have the pleasure of meeting you in Chicago next September, I am,

Very respectfully yours,

V. T. ATKINSON,
*State Secretary, U.S.V.M.A.*

Dr. Atkinson's letter is so good that any comments of mine upon it would only take away from its effectiveness.

In New Jersey, Dr. Autenreith, of Jersey City, thinks the law fails to be of any practical benefit to the profession at large, as its provisions are too vague and indefinite. The most hopeful feature about it, to him, is that the legislators of the State do recognize the fact that in time the existing laws may prove a nucleus for the development of further legislation, which may prove more useful and efficient in the advancement of the profession in that State. So far the New Jersey law, which was passed two years ago "has proven to be of no special benefit, though, perhaps, it may have inspired more confidence on the part of the laity in the qualified veterinarian, to such as know of his existence."

Dr. Kooker, writing for Pennsylvania, says that the law in that State has been properly enforced in only about half the counties, the prothonotaries in the other counties not having been particular as to who registered, or how they registered. The veterinary associations in Pennsylvania have brought several suits to bring about the observance of the law, most of which they have lost, and some of which have been appealed to the Supreme Court. He says that in his State the profession is strong, and has influential men in every county, and can get any proper law passed that they ask for. The trouble with the present law is that it was drawn up by veterinarians, when it should have been by lawyers, and he concludes that it is about as wise for veterinarians to act as lawyers as it would be for lawyers to act as veterinarians.

In New York State, judging from what Dr. Coates has to say, I should think the law worked better. As to the standing of the profession there, he says that in New York City the public are educated up to the point where they appreciate the qualified veterinarian, and in the rest of the State a man's standing in the community and success in obtaining practice depend largely on who he is and his social standing.

It seems to me that the legislation so far obtained is far from satisfactory. It legalizes a lot of ignorant quacks, some of whom can only make their marks when called upon to register in the county clerk's office, and that they are going to be a good many years in dying off before the coming graduates will be benefited. It recognizes a lot of mountebanks, who were much more easily ignored without laws of this class than they are with them. Furthermore, I believe that in this country anybody has a right to employ whom he sees fit to do anything, and that if an individual wishes to employ a quack to do his work, he has a perfect right to do so. It seems to be the modern idea that legislation of some sort must be a universal panacea for every ill, whereas we have more silly laws on our statute books than any country on the face of the earth, and most of them are either not half enforced, or else are dead letters altogether. The American people (not including the politicians, who make the laws) are a pretty level-headed lot, and in time will appreciate the distinction between the educated veterinarian and the quack horse doctor, and also between a veterinarian with an education and a graduate of a so-called veterinary school, who can scarcely read or write.

The following letter from Dr. John A. McLaughlin, of Providence, R. I., is so good and expresses so exactly what I think, that I take the liberty of reading it.

PROVIDENCE, R. I., July 8th, 1890.

Dr. AUSTIN PETERS:

DEAR DOCTOR: In answer to your note, allow me to state that there are no laws, as far as I am aware, protecting the veterinary surgeon. If there are, they are a dead letter.

As to the standing of the veterinary profession, it stands better with the public than it does with its own members; in other words, the public are willing, if not exactly anxious, to receive us at our own worth, from which I have but one suggestion—that we need *improvement* more than legislation; that we will benefit our profession more if we will put our endeavors in our own improvement than in demanding or begging legislation to make a veterinary "trust."

The public are intelligent enough to judge between the capable and the incapable man. If legislation were had to the effect that no citizen could employ any but a graduated veterinary surgeon, I believe that it would do more injury than good. The people here would kick, and kick hard!

I look upon the fact that the citizen of the United States is entrusted with more important matters—electing the president, for example—than choosing a veterinary surgeon to treat his animals. We want the best of everything, if we can afford it; and if we cannot, we want the best we can afford. As it is in everything else as I believe it is in veterinary matters. The public are willing to receive us, and give us our proper sphere, but I am afraid we fall just a little below their ideal, and I doubt if legislation will bring us one bit nearer to it. Respectfully,

JOHN A. MCLAUGHLIN.

On the other hand, there are legislative matters to which we migh much better give our attention, and compel the public and the government, both local and general, to appreciate the difference between the well-educated veterinarian and the ignorant empiric. Instead of endeavoring to pass laws to prevent someone hiring an ignoramus, if he wants to, we should devote our untiring energies to striving to secure a kind of legislation which, to my mind, would benefit us as individuals very much more, as well as the profession and the public. I refer to laws which would define, first, what an educated veterinarian is, and then forbid any veterinary testimony given in courts of law being considered as expert, except it be given by a properly-educated veterinary surgeon. I would also have it enacted that every State and city board of health have for one of its members a qualified veterinarian, and that such only should be employed for inspecting the animals at abattoirs, meats offered for sale in cities where a meat inspector is employed, and that a system of dairy inspection to secure a healthy milk supply be inaugurated in all States, and that the dairy inspectors must be educated veterinarians. I would also have it enacted that when the officials of a State, city or town employ a veterinary surgeon, that he must be a legally-qualified man. By thus compelling official recognition, the public could be more rapidly educated up to distinguish between the educated man and the quack, than in any other way.

In my own city of Boston for instance, the seat of culture and learning, where one might expect better things, you will find a veterinary school that you never even heard of; it has but one graduate and only three professors. The triumvirate composing the faculty is the Boston Board of Fire Commissioners, and the graduate who drives around with "V. S." worked in gold letters on his coat collar was formerly a hose man. The records say that the fire commissioners used formerly to employ a veterinary surgeon when they required one; but the veterinary requirements of the department as the city grew became so great that the office of veterinary surgeon was established, and a member of a certain hose company promoted to fill the position. If any public office requiring a young doctor were filled in such a way there would be a grand hue and cry; but as it is only a veterinary surgeon, it makes little difference who does the work. The emoluments of the position are probably not enough to cause a pang of envy to shoot through anyone's heart. I merely mention it as an instance. For a year past the Boston Board of Health has employed a qualified veterinarian as inspector as the Brighton Abattoir, which is a step in the right direction; but as yet we have no qualified inspector of meat in the city, and no dairy inspector exists at all, except that milk sold in Massachusetts has to come up to a certain chemical standard, thirteen per cent. of total solids; yet the milk is consumed without any knowledge of the health or sanitary surroundings of the cows producing it. Boston is ahead of most other large cities in this country in having a properly qualified veterinarian as inspector at the abattoir; in other respects she is much the same, and in other ways there are a few cities in advance of the rest in having veterinarians appointed to the board of health—for example, New York, Brooklyn and Jersey City. I have taken Boston as an example because living there I know the state of affairs there.

The recognition which we most demand from the general government just now is the organization of a proper army veterinary corps to place army veterinary surgeons in this country on an equality with commissioned officers, and with their confreres in every other country on the face of the globe having any claim to civilization. I will not say any more upon this matter, as I suppose there will be a report from the Committee on Army Legislation dealing with this subject in detail.

With regard to the standing of the profession in various parts of the country, I have incidentally received quite a good deal of information from the assistant State secretaries in response to my circulars. I find that veterinarians have the best standing in the communities where qualified men have been longest known, for example, Boston, New York City, and cities around New York; outside of these centres the West is the portion of the country that shows the greatest appreciation of veterinary science. With their great live stock interests as their principal sources of wealth, it is the Western States which have appointed State veterinarians and taken the most active steps to pass laws for the eradication and suppression of contagious animal diseases; and it is in the South where the greatest lack of progress is shown.

In Missouri the law is particularly good, because it first defines that the State veterinarian must be a graduate of a reputable veterinary college, besides which he must present good evidence to the trustees of the State Agricultural College that he has a good practical and theoretical knowledge of the diseases of animals before he can receive his appointment. If he cannot perform all his duties alone he can appoint deputies in various parts of the State to act as assistant veterinarians; and the law provides that they also must be competent and gradnates of veterinary colleges. This is a fitting tribute to the veterinary graduates that every State should be made to pay. This law provides for dealing with all infectious animal diseases, so that under it the State veterinarian can take measures to suppress tuberculosis and actinomycosis as well as other communicable maladies. The chief objection is the amount of salaries. I think $2500 a year is too small a salary for a State veterinarian, and I know $5.00 a day and expenses will not procure any very heavy timber to act in the capacity of deputies.

Taking one section of the country after another, judging from the letters I have received, and being as brief as possible, I find that in New Hampshire the people appreciate the usefulness of veterinarians, but do not yet readily distinguish between the graduate and quack. In Massachusetts the profession stands for the most part well, especially in Boston. Dr. McLaughlin's letter, which I have read, speaks for Rhode Island; and in Connecticut I find that the veterinary surgeon is constantly gaining in the esteem and confidence of people around the cities where his practice is, although he is little known or understood in the rural districts. Going outside of New England, I have already spoken of New York and New Jersey; in Pennsylvania the profession advances in public esteem by means of the veterinary department of the University of Pennsylvania and the veterinary association, and by the influence of the "JOURNAL OF COMPARATIVE MEDICINE," published in Philadelphia. From Maryland I learn that in and around Baltimore at least, veterinarians for the most part stand well with the public, and that they are recognized by the medical profession. Going further South, I find that in the District of Columbia and Virginia, the qualified man and quack stand on an equal footing, except that the one can demonstrate to the people that he is better than the other; South Carolina contains but two qualified veterinarians, but the one who wrote to me does not complain of his lot at all, always being treated considerately and courteously. Judging from a letter from Savannah, Georgia is much less of a veterinary paradise; the veterinarian is not only unprotected by law, but if a case dies he may be sued for damages by the owner of the patient; there are also no laws for the suppression of contagious animal diseases, and the people do not appreciate the graduate any more than a quack. From New Orleans I learn that the people appreciate the difference between qualified and unqualified men, by employing in the majority of cases the former.

In States North of a line drawn East and West through the Ohio River, and West of New York State, the profession advances rapidly, and the people appreciate it and make laws for the preservation of their live stock interests; while South of that, on the whole, a rather opposite state of affairs exists; this is due largely to the people as influenced by climatic and hereditary influences.

Other matters pertaining to the advancement of the profession could be dwelt upon in this report, but it does not seem best to continue it to too great length. In conclusion, I wish to express my hearty thanks to my fellow members on this committee, and to the assistant State secretaries who answered my circular, for the very able and kindly assistance they have rendered me.

AUSTIN PETERS, M.R.C.V.S.,

Secretary Hoskins—Dr. Peters has very kindly written to the officers of the association a letter containing a list of those State secretaries appointed in the past who have refused to answer communications sent them.

The list includes Dakota, Maine, Minneapolis, Utah, and Vermont.

Dr. Eves (Delaware)—Mr. President, I answered the letter referred to some time ago. I received one a few days ago which I did not answer.

Secretary Hoskins—I will very gladly scratch your name off.

President Michener—According to the programme, the discussion of the several papers is reserved until all of them have been read. Accordingly, we will now receive the report of this committee and lay it on the table until the others have been read or discuss it now, as you desire.

Dr. Griffin—I move you that the papers be discussed as read. Seconded.

Dr. Trombower—Mr. President, there are a number of committees to report, and I fear if we discuss each as submitted we will not get through very easily. Therefore I will oppose the motion, as I believe it will be better to take up the discussion after they have all been read.

Dr. Clement—I think it will take no longer to discuss them now than later on.

Dr. Faville—In that respect Dr. Clement is an interested speaker. I agree with Dr. Trombower and think it better to receive all the papers and discuss them as we see fit afterwards. The motion being put to a vote was lost and the discussion deferred.

President Michener—I will next call for the report of the Treasurer.

Treasurer Robertson submjtted his report as follows:

| | |
|---|---|
| In bank, September 15th, 1890 | $694 18 |
| Received from Secretary | 51 25 |
| | $745 43 |
| Paid Secretary deficiency, Brooklyn dinner account | 42 00 |
| Balance | $703 44 |

Secretary Hoskins—Mr. President, I will simply report that I have the vouchers here for the inspection of the finance committee. I will report that expenses last year were $543.69. Receipts, initiation fees and dues for the year, $576.50. I would ask you to refer to the items here.

President Michener—I believe the members of the finance committee are absent, and I would appoint as members of the finance committee, Drs. William Dougherty, Robertson and Williams.

We will next receive the report of the committee on diseases.

Dr. A. W. Clement submitted the report of the committee on diseases as follows:

### REPORT OF THE COMMITTEE ON DISEASES.

During the past year a good deal has been published upon the investigation of infectious diseases. Much of the work shows evidence of thoroughness, while some of it is open to severe criticism.

The duties of such a committee as this are not very well defined, but it seems to us that a passing in review of some of the work which has been brought more prominently before the profession, if discussed in an impartial manner, may be of as much importance as the devotion of more energy to the collection of statistics.

The investigation of the infectious diseases is a slow and oftentimes tedious process. The observer must work methodically, and oftentimes under very great disadvantages.

The work has become so systematized, and so much is being done in this line, that to be hampered by the practical duties of life means, too often, failure in both, to him who would combine clinical medicine with the study of the etiology of disease.

The busy practitioner claims from the laboratory specialist, and his claim is a just one, that such accurate information shall be given him of the cause of disease, and the best remedies to combat such diseases, as are recognized by the more or less scientific classification of clinical medicine The public claim from the specialist such information as will enable them, if possible, to guard against or to control such diseases as affect a large number of individuals at one time. Such information, usually termed practical, is too often the only information thought to be of any value by the public, and, unfortunately, by a large number of the members of our profession. We are too apt to forget that the only practical information is that which is based upon well understood scientific facts—facts which are the result of patient observation—and the accurate wording of them.

It is not for one moment to be supposed that there are any members of this association present who are not fully in accord with the germ theory of infectious and miasmatic diseases, and that there is no possibility of the spontaneous origin of this class of diseases. A case of contagious pleuro-pneumonia must be preceded by a case of contagious pleuro-pneumonia. The same is true of tuberculosis, Texas fever, etc., etc. Now, the term germ must not be confounded with the term bacteria, for it has been very well demonstrated that other organisms than bacteria are capable of producing disease.

A well-established example of infection by organisms of a non-bacterial type is malaria in man, and, according to some authorities, Texas fever in cattle. Such diseases as the above are classed as miasmatic diseases; *i. e.*, diseases in which the infecting agent requires a part of its development to take place outside of the body, in contra distinction to those diseases due to bacterial infection, where the development is completed inside the body, and the infecting element is thrown off from the body in a condition capable of producing the same disease in another individual.

A great deal of attention is being given at present to those diseases in which there is very considerable evidence of their miasmatic origin. As already stated, the typical miasmatic disease in man is malaria. Its association with non-bacterial micro-organismal infection dates from the researches of Laveran, in Algiers, communicated to the Paris Academy of Medicine in 1881–1882, as also in a large work on the malarial origin of fevers in 1884. As characteristic elements of the blood of persons affected with malaria were found: 1. Pigmented bodies in the interior of red blood corpuscles, which underwent amaboid changes; 2. Crescentic pigmented bodies; 3. A pigmented flagellated organism.

These investigations have since been confirmed by several observers, among them Marchiafava and Culi, and by Dr. Councilman, of Baltimore, Md. In 1880, Veterinary Surgeon Griffith Evans described a very fatal disease in horses, mules and camels in India. He discovered a parasite in the blood during life which he first described as a spirillum, but afterward concluded it was a much higher organism. In 1885, Steel found the same parasite described by Evans, and regarded it as a true spirillum. From clinical observation he concluded that the disease was very closely related to recurrent fever of man. Both Steel and Evans found the disease readily communicable, either by injection or inoculation to dogs, horses, and mules. Cruikshank afterward determined the bodies found in the blood to be a flagellate organism belonging to the group of infusoria.

Burke, Nissum, Raymond and others have written much upon diseases of the malarial type in animals. Some interesting and apparently very accurate investigations have recently been made by Dr. Theobold Smith, of the Bureau of Animal Industry at Washington, D. C. The results of these investigations were published in a paper entitled "Preliminary Observations on the Micro-organism of Texas fever," read at the Brooklyn meeting of the American Public Health Association, October 23d, 1889. He says: "Southern cattle fever or Texas fever, as it is more popularly known, is an infectious disease of the malarial type." . . . "The infectious agent, bound to a particular locality, is only temporarily transferred by cattle to places free from permanent infection." He experimented upon some native cattle by placing them in the same enclosure with healthy cattle shipped from North Carolina. The experiment was performed at the experimental station in Washington. "The first death occurred in August." . . "Up to the last week in October, ten had succumbed to the disease and two recovered." . . . "There was also a continual increase or accumulation of the virus in the enclosure, for the animals placed on the grounds late in the season died after an exposure of but one-half to one-third the period which was necessary to destroy those exposed since early Summer." Investigations looking toward the bacterial origin of the disease resulted negatively. He found, however, "small round bodies, perhaps 1 m. in diameter, centrally or somewhat eccentrically situated in or upon many red-blood corpuscles, which stain fairly in an aqueous solution of methyl violet. They there resemble micrococci in size and form. Unstained they can be seen as pure transparent spaces in the corpuscles."

From these observations made in 1886, and from the negative result of his bacteriological work upon the disease in 1888, Smith came to the conclusion that the disease must be due to a blood-parasite, which for its investigation would need careful microscopical research. For this purpose cattle were brought to the experimental station as above stated. He says: "The intra-globular bodies observed in 1886 were found in all the ten fatal cases of Texas fever." "In fresh spleen pulps they are visible as round or oval, nearly colorless spots from ½ to 2 m. in diameter on the disk of the red corpuscles, and always somewhat eccentrically placid. Careful focusing leaves no doubt that they are within the body of the corpuscle." "The smaller forms then appear as deeply stained cocci, about ½ to 1 m. in diameter, situated within the unstained circle of the corpuscle. Occasionally the bodies are nearer two feet in diameter, and then the staining may be less dense. Besides the spherical forms, ovoid forms are not uncommon. These usually occur in pairs within the same red-blood corpuscle. A still rarer pear-shaped form is encountered in stained preparations of the blood. It is rounded at one pole, while the other is pointed and sometimes drawn out as a short filament." . . . "One other abnormal form found in the blood deserves mention. These dried cover-glass preparations are stained with Loefler's alkaline methylene blue. A few red corpuscles appear as if their surfaces had been dusted over with minute specks of coloring matter. Whether they are due to the anæmia, or whether they belong to the cycle of the parasite, remains to be determined experimentally."

As to the distribution of the parasites, Smith concludes that the circulating blood, as a rule, contains comparatively few. "They may be numerous in the liver and spleen, and almost absent in the blood of the right ventricle." . . . "They are somewhat more numerous in the spleen than in the liver.'" Three rabbits were inoculated with spleen pulp stirred up in salt solution without, however, producing any effect. No classification of the parasite has, as yet, been attempted.

The conclusions reached by the author are as follows: "It is essentially a blood disease. There is a continuous or paroxysmal distribution of red blood corpuscles, due to an intra-globular parasite; and the disease results mainly from the incapacity of the internal organs, primarily the liver, secondarily the spleen and kidneys, to transform and remove the waste products resulting from such destruction. In milder cases, the protracted anæmia, which results from the loss of corpuscles, may become the chief cause of exhaustion and death, even when the organs remain pervious and capable of carrying on their respective functions."

Other observers, namely, Billings, of Illinois, and Paquin, of Missouri, consider the disease-producing element to be a bacterium, and both claim to have produced the disease by inoculation. Billings classifies it as a strictly local infectious disease and that only. He, however, does not make use of the classification generally adopted. He describes an infectious disease as one which "*Invariably* finds its origin not in, but outside, of the animal organism, *i.e.*, in the earth, where its microbic cause develops under certain conditions of the climate and soil which offer favorable climatic and telluric influences to its development." The infectious diseases of Billings are the miasmatic diseases of generally accepted authorities. The germ which Billings believes to be the cause of Texas fever he describes as morphologically the same as the germ which he finds in swine plague. He says: "These two organisms are neither to be classed with micrococci or bacilli." He classifies them as bacteria. He describes them as having a "longitudinal diameter about twice that of their transverse diameter." . . . "They are ovoid." . . . "Their ends are rounded." Like the swine plague germ, he says, they are motile. Inoculation of cultures, he claims, produced the disease in cattle.

It will be seen that the observations of Smith and Billings are quite contradictory. With Smith the bacteriological investigations were negative, but he found intra-organismal blood infection closely related apparently to that found in malaria in man, which is claiming so much of the attention of scientists to-day. Billings claims to have positive bacteriological results, which, he says, he has proven by producing the disease by inoculation of other cattle with pure cultures. I am personally unacquainted with the organism which Billings describes; but through the kindness of Dr. Smith have seen the intra-organismal elements which he describes. These certainly look like the bodies found in the red blood corpuscles of persons suffering from malaria.

Paquin, of Missouri, has done considerable work upon this disease, the results of which were published in "THE JOURNAL OF COMPARATIVE MEDICINE AND VETERINARY ARCHIVES," vol. XI, Nos. 7 and 8. His work is certainly open to very severe criticism. His material was collected in a very loose manner, and under such conditions as to make the investigation of no scientific value. Organs can not be collected in several different places remote from the laboratory where the investigations are to be made, and kept free from post-mortem inspection, even though they be wrapped in cloths soaked in corrosive sublimate solution and immersed in glycerine. Yet Paquin says: "During my trips in Texas and the Indian Territory, September, 1888, I collected soils, manures, urines, ticks, livers, spleens, kidneys, bile, specimens from unborn calves, fodders, and waters from various sources on infected grounds. Later, Dr. M. Francis, of the Agriculture College and Experiment Station of Texas, furnished me with a great number of articles of the same order, and later still, Dr. Dinwiddie, of Arkansas, gathered several. 'The specimens of blood, bile and urine were nearly all sealed in glass tubes (pipettes) without being exposed to the air.' He describes several germs which he believes to be different forms of the same organism, and the cause of Texas fever. He inoculated cattle with a modified virus, though it is not very clear how he modifies it, and claims to be able to confer immunity against the disease to cattle so inoculated."

### TUBERCULOSIS.

This very extensive and fatal disease has claimed a large share of the attention of investigators. Dr. Harold C. Ernst, of Boston, read a very instructive paper before the Association of American Physicians, Washington, September 20th, 1889, entitled "How Far may a Cow be Tuberculous Before her Milk becomes Dangerous?" Dr. Peters was associated with Dr. Ernst in this work. "One hundred and seventeen sets of cover glasses were examined from as many different samples of milk. Of these specimens thus spoiled, twelve turned sour before the examination was completed. These samples were obtained from thirty-six different cows, all of them presenting more or less distinct signs of tuberculosis of the lungs or elsewhere, but none of them having marked signs of disease of the udder of any kind." . . . .

"Of these samples of milk there were found seventeen in which the bacilli of tuberculosis were present; that is to say, the actual virus was seen in 31.5 per cent. of the samples examined. These seventeen samples of milk came from ten different cows, showing a percentage of detected infectiousness of 27.7 per cent.

"Rabbits inoculated with milk from cow, undoubtedly tuberculous but presenting no udder lesions, resulted in an infection of 10.2 per cent.; in guinea pigs, 28.57 per cent.; calves, 40 per cent."

It was also shown that the milk was infectious by inoculation experiments in 50 per cent. of the cows from which the milk came.

The conclusions drawn by Dr. Ernst are:

"*First*, and emphatically, that the milk from cows affected with tuberculosis in any part of the body may contain the virus of the disease.

*Second*, That the virus is present whether there is disease of the udder or not.

*Third*, That there is no ground for the assertion that there must be a lesion of the udder before the milk can contain the infection of tuberculosis.

*Fourth*, That, on the contrary, the bacilli of tuberculosis are present and active in a very large proportion of cases in the milk of cows affected with tuberculosis, but with no discoverable lesions of the udder."

For the benefit of those who are inclined to belittle the importance of scientific investigations from a practical standpoint, it may be said that Professor Robert Koch made the statement before the International Medical Congress, in Berlin, last month, that as a result of his investigations upon tuberculosis, he had found a substance which has the power of promoting the growth of tubercle bacilli, not only in test tubes, but in the body of an animal. He says, "My experiments are not completed, and I can only say this much about them: that guinea-pigs, which, as is well known, are extraordinarily susceptible to tuberculosis, if exposed to the influence of this substance seem to react to the inoculation of tuberculous virus, and that in guinea-pigs suffering from general tuberculosis, even to a high degree, the morbid process can be brought completely to a standstill without the body being in any way injuriously affected."

I noticed in the telegraphic news from Berlin the other evening that Koch was about to experiment upon human beings affected with tuberculosis.

HOG CHOLERA AND SWINE PLAGUE.

You are all aware, doubtless, of the controversy which has been going on with regard to the etiology of certain diseases of swine. As to whether there are two distinct diseases of swine as described in the reports of the Bureau of Animal Industry, or as to whether there is but one disease, as contended by Billings, which he calls swine plague.

In December, 1889, Prof. Welch published a "preliminary report of investigations concerning the causation of hog cholera." Ithas been my good fortune to be associated with him in this work for the past two years, and I can, therefore, speak from experience in the matter.

The conclusion has been definitely reached that there are two separate and distinct diseases, the one known as hog cholera and the other known as swine plague. They are due to separate and distinct organisms, which differ morphologically in their behavior in culture media, and in their reaction when inoculated into animals. It will not be necessary to go into the details of differentiation, as they have been placed before the profession many times in the past few years. Suffice it to say that in this report, while differing in some points from the conclusions reached by the workers on this subject in the Bureau of Animal Industry, the recorded observations harmonized with the facts observed in the investigations of these gentlemen, as reported since the year 1885.

Examination of direct slab cultures from the spleen, sent by Dr. F. S. Billings, proved them to be, in nearly all instances, pure cultures of the hog cholera bacillus. Much confusion has resulted, in the opinion of these investigators, from Dr. Billings' attempt to identify this organism with that of schweineseuche.

PLEURO-PNEUMONIA CONTAGIOSA.

Nothing has been done lately, that I am aware of, in the investigation of this disease. The methods employed by the government—that of slaughtering diseased and exposed cattle, and the thorough disinfection of stables—have been so far successful that to-day the disease is thought to exist only in a very limited area around New York city.

Many more observations upon contagious and infectious diseases have been made, but the time will not permit your committee to report further upon them at this meeting. A. W. CLEMENT, V.S.,

*Chairman.*

President Michener—I will call upon Dr. Salmon for the report of the Prize Committee.

Dr. Salmon—Mr. President, there has been no papers presented to me as chairman of that committee, and consequently no prizes to be awarded.

President Michener—I will call for the report of the Special College Committee of which Dr. C. C. Lyford, of Minnesota, is chairman.

Dr. Lyford read the report of the Special College Committee, as follows:

REPORT OF THE SPECIAL COLLEGE COMMITTEE.

I sent letters to the different colleges with a request to answer a certain list of questions regarding the course of instruction they would suggest and the requirements, with a view of establishing a uniform course of instruction. I received from many of them simply their prospectus, and from others I received letters, which I will read:

MONTREAL, 5th September, 1890.

*Dear Lyford:*

In reply to yours of August 20th, I beg to say that I have repeatedly suggested what is now proposed, and, of course, will only be too happy to find that others are working in the same direction.

I have, however, given up any expectation of such a desirable arrangement being arrived at, in Canada, at least.

I beg to suggest that (*a*) every veterinary school should have a uniform matriculation, examination in writing, arithmetic, including vulgar fractions, reading aloud, dictation, English grammar, geography and Latin. (*b*) The curriculum should embrace anatomy, physiology, history, pathology and pathological anatomy, bacteriology and parasitic diseases.

Chemistry: Materia medica, veterinary medicine and surgery. Diseases of cattle, sheep and swine (botany or zoology optional).

(*c*) That the course should extend over three years at least—three sessions of six months each—which may be subdivided into six sessions of three months each.

(*d*) That a uniform written examination for the final or pass examination be also adopted.

(*e*) That uniform fees be charged for the different courses in all the colleges.

Of course, these are my own views only. Before I could subscribe to their adoption I would have to lay them before the faculty, but I have little doubt but they would be approved by them.

If Chicago and Toronto be got to join in such an arrangement, much good will have been accomplished.

Wishing the U. S. V. M. Association every success in such good work, and assuring you and them of every support and assistance I can give them in the accomplishment of it,

Yours very truly, D. MCEACHRAN, *Dean.*

TORONTO, September 13th, 1890.

*My Dear Sir:*

I have just returned from England. In reply to yours of the 1st, I have to state that I do not object to a three-session course, if all the principal colleges do the same, and carry it faithfully out.

I think compulsory practice under a qualified veterinary surgeon during the *greater part* of the *Summer vacation* is preferable to a *Summer session.* In Britain a Summer session is done away.

You will notice, from Annual Announcement sent, that students are either required to pass an entrance examination, or present satisfactory testimonials as to education.

In some instances, if a candidate fails, he is allowed (the same as in Edinburgh) to undergo a second examination during the following Summer. This, in most instances, has done well; in others, however, the candidates, finding they could not pass their examination, have gone to other colleges, and duly graduated after attending one session.

I have understood that the American Veterinary College curriculum requires three sessions. I find, however, if a student attends one session here, he can graduate after attending another session at that college.

Mr. Mitchell, of Indiana, who entered our college beginning of session 1888–9, went to American Veterinary College in October last, and graduated in February. I merely mention this fact (which Prof. Liautard can explain); I am not doing so in a spirit of fault-finding, as I highly esteem both Prof. Liautard and Mr. Mitchell—the former for what he has done for our profession, and the latter as an excellent student and a worthy young man.

One board of examiners is impracticable. In Canada every province has control of all educational matters. You are also aware that some agricultural colleges in your country are granting veterinary degrees, which is quite right when that degree is merely a branch of agricultural science; but such degrees are given to men who intend following the veterinary profession as a means of livelihood. Agricultural colleges are of great value, but I do not know any of them that have proper facilities for the thorough teaching of our profession.

I am sorry I cannot be present at your meeting. With kind regards,

Yours in haste,

ANDREW SMITH.

MR. C. C. LYFORD, V.S.,

Tremont House, Chicago, Ill.

PHILADELPHIA, September 13th, 1890.

*Dr. C. C. Lyford, Chairman.*

MY DEAR SIR: In reply to your letter of August 20th, I desire to say that our faculty is in hearty accord with the movement to have the veterinary colleges of this country adopt a common stand of requirements for admission. In this, however, we might add that we could not consent (for our own school) to the adoption of a standard lower than that already adopted by us.

I very much regret that duties here will prevent my being with you at the meeting in Chicago; however, one of our faculty, Dr. Zuill, will be in attendance.

I remain very sincerely yours,

JOHN MARSHALL.

NEW YORK, September 13th, 1890.

*My Dear Doctor:*

Yours of the 7th at hand.

I favor very much a curriculum requiring a three-years' course—a preliminary examination in such branches as one would receive in a common school, and a board of examiners, composed of a teacher from each of the various veterinary colleges, that teacher to be delegated by the faculty or trustees of the school from which he came.

Yours very truly,

To DR. C. C. LYFORD, HARRY D. GILL.
*Chairman.*

NEW YORK, August 25th, 1890.

*Dr. C. C. Lyford, Chairman.*

*Dear Sir:* In reply to your note of the 20th, I beg to inform you that I have mailed you this A. M. one of our Announcements for 1890–91, where, I hope, you will find the information desired. I send you this, as I do not exactly see what you desire, and thought perhaps the Announcement would tell you all. I remain yours very truly,

A. LIAUTARD.

NEW YORK, September 10th, 1890.

*Dear Doctor:*

Yours is just received. As the questions you ask me to answer are part of statements which I intend to present the association, I will be pleased to offer them to you on Monday or Tuesday, as I shall be then in Chicago. I can, however, say (1) I am in favor of a three-years' course, and (2) I believe I am the *first* who suggested and recommended the establishment of a board of examiners. The minutes of the association and the back numbers of the *Review* will show my claim to priority. But more later on. Yours truly, A. LIAUTARD.

ROBITAILLE, P. Q., August 29th, 1890.

*My Dear Sir:*

Your letter of August 10th has just been received by me here. The U. S. Veterinary Association has had for some years now a committee upon "Single Standard" for the various schools, to whose chairman I have, from time to time, written my ideas concerning the matter; therefore a communication of length from me to you will not be necessary at this time. It would, in my opinion, be very much to the advantage of our profession if all of the American schools advanced their standard to that, let us say, of Montreal. That they will do so, I do not believe. Our art and science *cannot* advance so long as the great majority of its practitioners begin life with eleven months, or less, of study in ungraded schools; and we are the only veterinary profession which asks our public to believe that there is so little in our art. Yours truly,

PROFESSOR C. C. LYFORD. CHARLES P. LYMAN.

Minneapolis, Minn.

CHICAGO, August 27th, 1890.

*Prof. C. C. Lyford, N. W. Veterinary College, Minneapolis, Minn.:*

*Dear Sir:* Before receiving your letter we mailed you our prospectus for the coming session. We do not know if this has given you the information required. If not, please state more fully what you want—whether graduation or matriculation—and oblige, Yours truly,

R. J. WITHERS,
*Presidenl.*

President Michener—The next in order will be the reception of the report of the Committee on Army Legislation.

Dr. R. S. Huidekoper, chairman of the Committee on Army Legislation, made the following report:

REPORT OF THE COMMITTEE ON ARMY LEGISLATION.

Your committee has the honor to submit the following report:

After our appointment as a committee on army legislation, at the meeting of the United States Veterinary Medical Association last year, we collected, from all sources, copies of the various bills which had in past years been submitted to Congress on the subject of army veterinary legislation, and were furnished with copies of several new bills containing more or less changes from previous ones. From these we made a digest and outlined a bill asking for the organization of a veterinary department, to be added to the other departments of the United States Army. We asked in this bill for the organization of a corps of veterinarians, to consist of one chief veterinarian, with the rank of colonel; one veterinarian, with the rank of lieutenant-colonel; one veterinarian, with the rank of major; ten veterinarians, with the rank of captain, and twenty veterinarians, with the rank of lieutenant.

This draft of the bill we submitted to General Schofield, to the Surgeon-general, to officers of the staff department who would be especially interested in it, and to the officers at a number of military posts both in the East and West, by whom the matter was courteously considered; and we received advice both from older officers, high in rank, and from younger officers, who are at present actively engaged in the practical details of the cavalry and artillery. After mature deliberation as to what was needed, and careful consideration of the limit which we could expect to obtain from Congress, in numbers and rank, for the new corps, we submitted a modified draft of the bill to General Schofield, who still further modified it into a form which would be acceptable to him, and this we gave to the Honorable H. H. Bingham, of Pennsylvania, who kindly introduced it into the House of Representatives, where it was read twice, referred to the Committee on Military Affairs, and ordered to be printed on January 6th, 1890, Number H. R. 3912, 51st Congress, 1st Session.

The bill read as follows:[1]

[1] See February number JOURNAL OF COMPARATIVE MEDICINE AND VETERINARY ARCHIVES, page 113.

In the next few weeks we made a number of visits to Washington, and saw individually each member of the Committee on Military Affairs in the House of Representatives. At about that time Senator J. Donald Cameron, of Pennsylvania, introduced the same bill into the Senate. We obtained permission and an invitation from the chairman of the Committee on Military Affairs of the House, for the commander of the cavalry post at Fort Myer, other cavalry officers, a representative of the medical department, a former staff officer of General Sheridan's—who, under General Sheridan, had investigated the subject of veterinary service—and other officers to appear before the committee, which they did, stating in distinct terms the necessity for the organization of such a service. These officers, unquestionably, exercised considerable weight with the committee. The bill was referred by the Committee on Military Affairs to the War Department for its consideration, and, to our surprise, was returned with an endorsement of the Major-general commanding the army, stating that while improved veterinary service was undoubtedly needed, he questioned the advisability, at the present time, of forming a new department, and adding so many commissioned officers to the roster of the army. Your committee called upon General Schofield, and, at its solicitation, the General was also called upon by members of the Committee on Military Affairs, when we learned that General Schofield was not, as the endorsement seemed to indicate, opposed to the establishment of a more efficient veterinary service, but that, with the large number of other demands which were being made in Congress for the army, he deemed it unadvisable to attempt a service on so large a scale at present, and, by his advice, a new bill was framed, submitted to him, and, with his approval, was introduced in the House of Representatives on March 26th, 1890, by General Joseph Wheeler, of Alabama, under the number H. R. 8638, 51st Congress, 1st Session.

It reads as follows:

A Bill to Provide for a More Efficient Veterinary Service.

*Be it enacted by the Senate and House of Representatives of the United States of America in Congress assembled:*

That there shall be, and hereby is, added to the quartermaster's department of the United States Army a veterinary division, which shall consist of one chief veterinarian, with the rank, pay and allowances of a major of cavalry, who may be appointed by the President of the United States, by selection, with and by the consent of the Senate; four veterinarians, with the rank, pay and allowance of first lieutenants of cavalry, and ten assistant veterinarians, with the rank, pay and allowances of second lieutenants of cavalry.

Section 2. That as soon as practicable, after the passage of this act, the President of the United States may appoint a veterinary medical examining board, which shall consist of the chief veterinarian, two officers of the quartermaster's department, and two officers of the medical department, whose duty it shall be to examine such candidates as shall present themselves for examination for appointment in the veterinary division, and shall report and certify to the Secretary of War the names of the candidates who shall have passed the highest examination satisfactory to said board.

SEC. 3. That upon the receipt from the said examining board of the certificates of the candidates who shall have passed the highest satisfactory examination, the President of the United States may appoint to the various offices junior to the chief veterinarian said successful candidates, the said appointees to take rank according to the order of merit certified by said examining board, not to exceed the number provided for in Section 1 of this act.

SEC. 4. That all veterinary surgeons of the United States army who, at the passage of this act shall be in service, may be granted three months' leave of absence with full pay, for the purpose of preparing themselves for examination.

SEC. 5. That the Secretary of War shall hereafter appoint from time to time a veterinary examining board, which shall consist of the chief veterinarian and two veterinarians of the United States army veterinary division, to examine candidates for the position of assistant veterinarians' with the rank of second lieutenant and for promotion in the division.

SEC. 6. That promotion below the rank of field officer shall be by seniority, but no officer of the division shall be entitled to promotion thereby until he shall have been examined and approved by a veterinary examining board; and if any such officer fail on examination he shall be suspended from promotion for one year, when he shall be re-examined before a like board, and in case of failure on such re-examination he shall be discharged from the service.

SEC. 7. That officers of the veterinary division shall not be eligible for promotion other than in that division.

SEC. 8. That any of the present veterinary surgeons who shall fail to pass the examination required by Section 2 in this act shall be discharged with one year's pay.

This bill was returned to the committee with the approval of the War Department, but was placed upon the calendar of the House by the committee, with still further modifications, which consist essentially of the reduction of the rank of chief veterinarian from that of major to that of captain of cavalry, and the elimination of the word "rank" in connection with the position of the other officers. In the Committee on Military Affairs of the Senate, the matter was under consideration when the modification took place in the House of Representatives, and your committee then deemed it unadvisable to spend further time in the Senate until the matter was settled in the House.

In a letter from General Joseph Wheeler, under date of August 26th, your committee is informed "that the veterinary bill has been reported favorably and will probably soon come up in the House, and it is understood that the Military Committee will have a day, and that we expect to get the bill up." Your committee is assured that the word "rank" will be reinserted by the Military Committee in the Senate, and that after a conference committee the change will be accepted by the House.

At an early period in the Winter, and at various times later on, we were informed by officers at the Army Headquarters, by Senators and by Congressmen who were interested in this legislation, that they were in constant receipt of letters opposing the action of your committee, and requesting their influence to check any action being taken on the bills introduced in behalf of the United States Veterinary Medical Association ; also, from time to time, other bills, looking toward the organization of a veterinary corps in the army, were introduced both in the Senate and the House of Representatives. Upon investigation, your committee found that the other bills introduced in the Senate and in the House of Representatives, and the opposition to the action of the committee of the United States Veterinary Medical Association, emanated from the same sources. These sources were principally veterinarians now in the employ of the army, some as veterinarians in the cavalry, and others as contract veterinarians in the other arms of the service. Whether this bill passes the present Congress and becomes a law or not, we are satisfied that very great progress has been made. A live interest has been aroused in the minds of the army officers themselves that an improvement in the system of veterinary service is demanded in the interests of the army itself. Members of Congress have learned that it is needed, and we have the precedent of having carried a bill through the Committee on Military Affairs and placed it on the calendar. For the guidance of your future committees, we feel entitled, as the result of our hard labor, to be competent to judge of what is needed, and we presume to dictate certain courses which must be pursued if any success is to be looked for.

1st. A bill for an army veterinary service mnst have the approval of the War Department before it will be considered in committee.

2d. The first service established must be a small one, with officers of moderate rank. or it will otherwise incur the natural jealousies of officers of other arms who would be outranked at small posts.

3d. No service will be established which does not demand examination as one of the qualifications for entering it.

4th. The misfortune of any one or more veterinarians previously employed by the government, who might be thrown out by an examination, must not be allowed to weigh one atom as against the good of the whole profession and the better service for the army which could be rendered by veterinarians endowed with the authority with which a commission as an officer would invest them.

Your committee has performed its work with regard only to the good of the profession at large ; it would like to have done more, but every matter had to be looked to in person, and numerous visits to Washington entailed not only loss of time, but also considerable expense. We are indebted to a member of the Association, who desires to withhold his name, for a check which defrayed the expense of one visit.

Respectfully,

RUSH S. HUIDEKOPER, *Chairman*,
DANIEL LEMAY,
COOPER CURTICE, *Committee*.

