# Correction

**Published:** 1890-12

**Authors:** 


CORRECTION.

November number, page 669, line 7, read Actinomycosis for Tuberculosis.

